# Premenstrual symptoms and remedies practiced by Malaysian women attending a rural primary care clinic

**DOI:** 10.4102/phcfm.v1i1.18

**Published:** 2009-05-08

**Authors:** Khairani Omar, Siti S. Mohsin, Leelavathi Muthupalaniappen, Idayu B. Idris, Rahmah M. Amin, Khadijah Shamsudin

**Affiliations:** 1 Department of Family Medicine, Universiti Kebangsaan, Malaysia; 2 Outpatient Department, Hospital Kuala Lumpur, Malaysia; 3 Department of Community Health, Universiti Kebangsaan, Malaysia

**Keywords:** premenstrual symptoms, premenstrual syndrome, reproductive age women, remedies, primary care

## Abstract

**Background:**

Premenstrual symptoms affect about 40% of women of reproductive age. In an effort to alleviate premenstrual symptoms, affected women practice various remedial approaches. The aim of this study was to assess the prevalence and severity of premenstrual symptoms experienced by women, the associated factors and the remedial approaches practiced by them.

**Method:**

This was a cross-sectional study conducted at a rural primary care clinic situated in Hulu Langat, Malaysia. All women of reproductive age (18 to 44 years old) attending the clinic during the study period and who fit the selection criteria were included. Premenstrual symptoms and severity were assessed using a self-report questionnaire, the Shortened Premenstrual Assessment Form (SPAF). It consists of 10 items that measure changes in mood, behaviour and physical symptoms. The respondents were also asked if they had used any remedy to relieve their symptoms.

**Results:**

A total of 158 women were included in the study. The majority of the respondents were Malay (70.3%), followed by Indian (16.5%) and Chinese (10.8%) women. About 75% of the women experienced at least one of the premenstrual symptoms. Approximately 7% of them reported experiencing severe symptoms in all three subscales of the SPAF. The frequently reported symptoms were body ache (75.3%), abdominal pain (75.3%), irritable feeling (63.9%) and breast discomfort (61.4%). The symptom score was higher among Malay women (p = 0.034), and those with a higher household income (p = 0.037) and higher educational level (p = 0.01). There was no significant association between premenstrual symptoms and age, marital status, menstrual cycle and age of menarche. The common remedies used were vitamins (19%), a healthy diet (15.8%) and analgesics (13.3%). Approximately 60% of the women did not use any remedy to reduce their premenstrual symptoms.

**Conclusion:**

Premenstrual symptoms were common among women attending the clinic. The symptoms affect them significantly both physically and emotionally. Thus, it is essential for primary care providers to take an active role in identifying, educating and managing premenstrual symptoms among women.

## INTRODUCTION

Premenstrual syndrome (PMS) is characterised by the recurrence of certain physical, psychological and behavioural symptoms, beginning the week before menses and disappearing within a few days after the onset of menses.^1, 2, 3, 4^ The key diagnostic feature is that the symptoms must be absent in the time between the end of menstruation and ovulation. Premenstrual symptoms affect about 40% of women of reproductive age, with severe impairment occurring in approximately 5%, enough to impair their daily life and relationships.^1, 3, 5, 6, 7^ Most women of reproductive age have one or more emotional or physical symptoms, which are often mild, in the premenstrual phase of the menstrual cycle.^3^

The severe and predominantly psychological form of PMS is called premenstrual dysphoric disorder (PMDD).^2, 3, 8, 9^ The diagnosis of PMDD on the basis of DSM-IV stipulates (1) the presence of at least five luteal-phase symptoms, at least one of which must be a mood symptom (i.e., depressed mood, anxiety or tension, affected lability or persistent anger and irritability); (2) two cycles of daily charting to confirm the timing of symptoms; and (3) evidence of functional impairment. Finally, the symptoms must not be the exacerbation of another psychiatric condition.^10^ However, many women with clinically significant premenstrual symptoms do not meet full diagnostic criteria; they might not have a prominent mood symptoms as one of the five different symptoms required as a minimum by DSM-IV. The American College of Obstetrics and Gynecology (ACOG) has attempted to rectify this situation by defining moderate to severe PMS; the criteria are the presence of at least one psychological or physical symptom that causes significant impairment and that is confirmed by means of prospective ratings.^11^

The exact aetiology of PMS is not known.^2, 6^ It does not seem to be due to abnormal concentrations of sex steroids, but the symptoms are triggered by fluctuations in such hormones; some patients probably are more sensitive to such fluctuations. With respect to brain function, the transmitters serotonin and GABA have been implicated in the underlying mechanism. Treatments inhibiting ovulation, such as GnRH analogues, oestrogen and certain new oral contraceptives, effectively reduce the symptoms, as do treatment with selective serotonin reuptake inhibitors (SSRIs), which are regarded by some institutions as first-line agents in severely affected patients.^2, 3, 4, 5, 6, 8, 9, 12^

In an effort to alleviate premenstrual symptoms, affected women practiced various remedial approaches. Previous studies have examined many factors to elucidate the underlying ones contributing to this syndrome.^2, 3, 8, 12, 13^ These include lifestyle factors, affective state, marital status, dietary pattern, medication used, physical exercise, and socio-economic factors, and biological factors such as age and reproductive and menstrual history^.^. Women with premenstrual symptoms could improve their quality of life by understanding their body's cyclic changes, planning their lives and seeking treatment when they anticipate experiencing premenstrual symptoms.^14^ The aim of this study was to determine the prevalence and severity of premenstrual symptoms experienced by women attending a primary care clinic, the associated factors and the remedies used by them. The associated factors that were analysed were age, ethnicity, household income, educational level, marital status, age of menarche and duration of menses.

## METHOD

This was a cross-sectional study conducted at a rural primary care clinic situated in Hulu Langat district in Selangor, Malaysia. All women attending the clinic during the study period who fit the selection criteria were included in the study. The inclusion criteria included all women aged 18 to 44 years old who were registered as patients at the primary care clinic. Women who were pregnant, had early menopause or severe mental and physical disability were excluded from the study.

Premenstrual symptoms and severity were assessed using the Shortened Premenstrual Assessment Form (SPAF). SPAF is a self-report questionnaire developed by Halbreich et al.^15^ and its validity has been described.^9, 15, 16, 17^ It consists of 10 items, which measure changes in mood, behaviour and physical symptoms during the premenstrual period. The symptoms are divided into three subscales, namely symptoms that describe affect, water retention and pain. A total score is calculated based on the summing of positive response to each symptom. The severities of the symptoms were assessed using a rating scale of 1 to 6 based on changes in comparison to the non-premenstrual state. Scale 1 indicates ‘no change’, 2 – ‘minimal change’, 3 – ‘mild change’, 4 – moderate change, 5 – ‘severe change’ and 6 – ‘extreme change’. The respondents were also asked if they had practised any remedial approaches or taken any medication for the premenstrual symptoms.

The data was analysed using the Statistical Package for Social Sciences (SPSS) version 14. T-test, mean and standard deviation were used to analyse the data. A p value of < 0.05 and a confidence interval of 95% were taken as indicative of significant difference. Ethical approval was obtained from the Ministry of Health of Malaysia and the Research Committee of the Universiti Kebangsaan Malaysia.

## RESULTS

A total of 158 women agreed to participate in the study. The majority of the respondents were from the Malay ethnic group (70.3%), followed by Indian (16.5%) and Chinese (10.8%) women. Their ages ranged from 18 to 44 years, with a mean age of 30.7 years. More than half (73.4%) were married. Most of the respondents had a secondary education (91.8%), while 8.2% had tertiary education. Most (79.1%) of the respondents had a household income of less than RM 2000 a month (Table 1).

**TABLE 1:** Association between PMS score and demographic factors

VARIABLES (N = 158)	N	%	MEAN SYMPTOM SCORE ± SD	P VALUE
**ETHNIC GROUP**				**0.034**
Malay	111	70.3	28.21 ± 1.3	
Chinese	26	16.5	23.62 ± 9.3	
Indian	17	10.8	22.53 ± 8.2	
Other	4	2.5	19.50 ± 3.3	
**AGE GROUP**				**0.769**
18 – 24	41	25.9	26.68 ± 10.4	
25 – 34	61	38.6	27.34 ± 11.26	
35 – 44	56	35.4	25.66 ± 10.86	
**HOUSEHOLD INCOME**				**0.037**
< RM 1000	54	34.2	25.02 ± 9.9	
RM 1000 – RM 1999	71	44.9	25.70 ± 10.7	
RM 2000 – RM 2999	17	10.8	29.53 ± 10.2	
RM 3000 – RM 3999	16	10.1	32.94 ± 12.6	
**EDUCATIONAL LEVEL**				**0.01**
Secondary	145	91.8	25.76 ± 10.5	
Tertiary	13	8.2	36.31 ± 10.0	
**MARITAL STATUS**				**0.908**
Single/divorced/ widowed	42	26.5	26.91 ± 11.82	
Married	116	73.4	26.56 ± 10.48	
**AGE OF MENARCHE**				**0.743**
< 12 years old	70	44.3	26.00 ± 10.41	
> 13 years old	88	55.7	27.11 ± 11.16	
**DURATION OF MENSES**				**0.408**
≤ 6 days	76	48.1	25.13 ± 10.57	
≥ 7 days	82	51.9	28.00 ± 10.92	

**SYMPTOMS SCORE**		**Range**	**Mean score ± sD**	

**OVERALL**		**12–60**	**26.0 ± 4**	

The majority of the respondents (> 75%) experienced at least one of the premenstrual symptoms. Approximately 7% of the respondents reported experiencing severe or extreme premenstrual symptoms in all three subscales of the SPAF, namely affect, water retention and pain. This study showed that the symptoms score was higher among Malay women (p = 0.034) and those with a higher household income (p = 0.037) and higher educational level (p = 0.01). No significant association was found between premenstrual symptoms and age, marital status, duration of menses and age of menarche (Table 1).

The most frequently reported premenstrual symptoms were physical symptoms of body ache (75.3%) and abdominal pain (75.3%), followed by irritable feeling (63.9%) and breast discomfort (61.4%). The symptom score based on the Shortened Premenstrual Assessment Form (SPAF) ranged from 12 to 60, with a mean score of 26.0 ± 4 (minimum score = 10, maximum score = 60) (Table 2).

**TABLE 2:** Frequency and severity of premenstrual symptoms among respondents

SYMPTOMS (N = 158)	YES		MILD		MODERATE		SEVERE	
			
	n	%	n	%	n	%	n	%
Breast pain, enlargement or swelling	97	61.4	44	27.8	45	28.5	8	5.1
Outburst of irritability or bad temper	101	63.9	40	25.3	44	27.8	17	10.8
Body ache, joint pain or backache	119	75.3	41	25.9	55	34.8	23	14.6
Abdominal discomfort, heaviness or pain	119	75.3	39	24.7	55	34.8	25	15.8
Feeling bloated	70	44.3	22	13.9	34	21.5	14	8.9
Feeling stress	94	59.5	49	31.0	32	20.3	13	8.2
Feeling depressed	84	53.2	35	22.6	36	22.8	13	8.2
Weight changes (weight gain)	73	46.2	30	19.0	32	20.3	11	7.0
Oedema, swelling, puffiness or water retention	69	43.7	38	24.9	21	13.3	10	6.3
Cannot cope or overwhelmed by ordinary demands	69	43.7	16	10.1	39	24.7	14	8.9
Severe symptoms in all subscales (affect, water retention, pain)	11	7.0						

The majority of the women did not seek any remedy for their premenstrual symptoms. About 40% of the respondents reported that they had tried at least one type of remedy, and about 28.5% had used a combination of remedies. The most popular options for remedial approaches were vitamins (19%), followed by a healthy diet (15.8%) and analgesics (13.3%) (Table 3).

**TABLE 3:** Remedies used to alleviate PMS

REMEDIES N = 158	(N)	%
Did not seek treatment	94	59.5
Vitamins	30	19.0
Healthy diet	25	15.8
Analgesics	21	13.3
Exercises	13	8.2
Herbal	12	7.6
Hormonal (OCP)	9	5.7
Combination of remedies	45	28.5

## DISCUSSION

The results of this study indicate that premenstrual symptoms (PMS) are common among women attending the primary care clinic, since the majority of the respondents (> 75%) experienced at least one of the premenstrual symptoms. The mean premenstrual symptom score was 26.0 + 4 (range 12-60), which is similar to that in several previous studies in other countries.^14, 16, 18, 19^ This implied that most of the women experienced mild premenstrual symptoms. Primary care providers should screen women attending the clinic for PMS and educate them regarding the symptoms. Although many women experienced mild symptoms, it is beneficial for the affected women to seek treatment and manage the symptoms to improve their quality of life.

Approximately 7% of the respondents reported experiencing severe premenstrual symptoms that impaired their daily life functions and relationships. This finding is similar to previous studies, which reported that 5 to 8% of women have moderate to severe symptoms that are associated with substantial distress or functional impairment.^1, 3, 5, 7^ Those women who experience moderate to severe premenstrual symptoms require further assessment to determine if they have premenstrual dysphoric disorder. Their quality of life could be improved, since significant advances have been made in the pharmacological therapy for PMDD. SSRIs have been shown in studies to be effective in relieving distressing mood symptoms and improving social function in most patients.^2, 3, 4, 5, 6, 8, 9, 12^

The commonly reported premenstrual symptoms were mainly physical symptoms, namely backache and joint pains, abdominal pain and breast pain. However, the emotions of the women were also significantly affected, with more than half of the respondents reported being irritable (64%) and feeling depressed (53%). Approximately 44% of the respondents reported that they could not cope or were overwhelmed by ordinary demands during the premenstrual period. This implies that the premenstrual symptoms significantly affected the women physically and emotionally. These results are similar to the findings of other studies in many countries.^7, 9, 14, 18, 19, 20^

In this study, premenstrual symptoms were reported more frequently by the Malay women than by women from the other ethnic groups. This could be due to the disproportionate study sample, where the majority of the respondents were Malay. Whether this reflects a specific biological, social or lifestyle characteristic remains to be determined. Women from the higher income group and those with higher educational levels reported a higher premenstrual symptom score. However, some other studies have described that better educated women and those from a higher income group were less likely to report PMS.^14, 20^ The results of this study can be explained by the local cultural behaviour, whereby women from the lower socio-economic community are more reserved about discussing PMS, and may regard it as a normal physiological event.

Approximately 40% of the respondents had tried at least one type of remedy for their premenstrual symptoms. The common treatments reported by the respondents were vitamin supplements, a healthy diet, analgesics and exercise. The findings of this study suggest that premenstrual symptoms affect a significant proportion of the women, which prompts them to seek various treatments.

Only about 19% of the women took prescribed treatment from health professionals, namely analgesics (13.3%) and hormonal pills (5.7%). Others took either supplementary medicine, non-pharmacological treatment or did not seek treatment (60%). This could be contributed to the mild symptoms that they experienced, meaning that they felt it to be unnecessary to seek help from health professionals, or that it was due to the socio-cultural behaviour of the community. However, there are many ways to alleviate even mild symptoms of PMS, and thus improving their help-seeking behaviour would be beneficial. Lifestyle modifications and exercise are recommended for all women with PMS and may be all that is needed to treat mild to moderate symptoms.^5, 9, 12^

Approximately 16% of the women followed a healthy diet to relieve their premenstrual symptoms. Premenstrual dietary modification, such as the restriction of salt use, has been proposed to alleviate symptoms of fluid retention, weight gain and breast swelling.^1, 7, 14, 21^ Caffeine restriction is also recommended for women with symptoms of premenstrual irritability and insomnia.^1^ Consuming frequent small meals and complex carbohydrates may be helpful to those women who typically engage in premenstrual food binges.^1, 7, 22, 23^

Exercise was used by 8.2% of the women to relieve PMS. Several studies have suggested that PMS would be attenuated by engaging in exercises.^1, 7, 13, 14, 23^ The positive effects of moderate exercise on mood and general health are well documented.^1, 7, 13, 14, 23^ Women engaging in moderate aerobic exercise at least three times a week have significantly fewer premenstrual symptoms than sedentary women.^24, 25^

Approximately 19% of the women took vitamins to reduce PMS. Vitamin B has been reported to have a significant effect in relieving PMS.^23, 26^ Meta-analysis concludes that doses of 50 to 100 mg elemental vitamin B6 are also significantly better than a placebo in relieving PMS.^26^ Few trials had documented a significant decrease in depression, water retention, pain, fatigue and insomnia in women who received 1 200 mg elemental calcium per day.^14, 16^ Evening primrose oil, a prostaglandin precursor, has been reported to relieve depression and mastalgia and to improve the global score of PMS.^1, 14^

Analgesics were used by 13.3% of the women. Most NSAIDs are effective in alleviating a wide range of PMS symptoms, but they do not appear to improve mastalgia.^7, 14, 27^ About 5.7% of the women had taken hormonal treatment, mainly oral contraceptives, to treat their PMS. Oral contraceptives would improve physical symptoms such as bloating, headaches, abdominal pain and breast tenderness.^28^ However, the pills do not appear to have a positive effect on mood symptoms.^14, 28, 29^

Future research should look into the women's understanding of PMS, their barriers to and reasons for not seeking help from healthcare providers, and the prevalence of premenstrual dysphoric disorder among those women who have extremely distressing emotional and behavioural symptoms. Advances have been made in the treatment of premenstrual dysphoric disorder, thus women with this disorder could be helped to improve their quality of life.^5, 6, 9, 12^

There are some limitations to this study. The use of structured questionnaires and not open-ended ones may lead to bias in reporting, especially when exploring new areas of human behaviour. Self-reporting also records data with a retrospective focus and the information may be subject to memory and reporting bias.

### Conclusion

The results of this study show that premenstrual symptoms are common among women attending the primary care clinic. Although many of the women had tried some remedies to alleviate premenstrual symptoms, the majority of them did not seek any treatment. Thus, it is essential for primary care health professionals to take an active role in identifying and managing premenstrual symptoms in these women and educating them.

Education can be given through various approaches such as pamphlets, posters, effective communication and facilitating supportive groups in the clinic, work place or community.
